# Oral language comprehension interventions in school-age children and adolescents with developmental language disorder: A systematic scoping review

**DOI:** 10.1177/23969415211010423

**Published:** 2021-05-24

**Authors:** Sirpa Tarvainen, Kaisa Launonen, Suvi Stolt

**Affiliations:** Department of Psychology and Logopedics, Unit of Logopedics, University of Helsinki, Helsinki, Finland

**Keywords:** Specific language impairment, speech and language therapy, focus of intervention, efficacy, level of evidence

## Abstract

**Background & aims:**

Difficulties understanding spoken language are associated with several social and academic risks in school-age children and adolescents with developmental language disorder (DLD). Still, interventions for this group have received little attention, and there are no reviews focusing on oral language comprehension interventions in school-age children and adolescents. The objective of this systematic scoping review was to identify interventions targeting oral language comprehension in school-age children and adolescents with DLD. Further, the aim was to examine the focus of intervention, efficacy, and level of evidence of the identified interventions. The present review is the second part of a larger search on oral language comprehension interventions. The first review examined the same factors in children 8 years and younger.

**Methods:**

A systematic scoping review of eight databases was conducted. Of the 2399 sourced articles, 12 met the inclusion criteria. Another 8 articles were identified through reference lists of sourced articles. In these 20 articles, containing 21 studies, 1661 children aged 5–16 years participated. The data were extracted and analysed, and the intervention focus, efficacy, and level of evidence were examined.

**Main contribution:** In the interventions intended for school-age children and adolescents with DLD, three intervention foci were identified that targeted aspects of language and language processing, as well as modifying the communicative environment. Of the included studies, 57% reported positive results, 14% reported mixed results, and 29% reported no effects on oral language comprehension. The level of evidence varied. One can have high confidence in the results of 19%, moderate in 38%, and indicative confidence in 43% of the included studies.

**Conclusions:**

Results of the present review suggest that there are a few interventions providing high confidence on the efficacy of improving oral language comprehension difficulties in school-age children and adolescents with DLD. Most interventions indicating efficacy provide moderate or indicative confidence in the results. More research with a high level of evidence is urgently needed. Most of the interventions indicating efficacy focused directly on language skills or modified the communicative environment. The results suggest that the therapy techniques focusing on improving language processing skills indicate efficacy only when they aim at compensating current language processing skills, not trying to improve them.

**Implications:** The findings on different therapy techniques, their focus of intervention, efficacy, and level of evidence provide information for clinical practice and direct future investigations in this sparsely researched topic.

## Introduction

Children who do not learn language like their peers, but who have persistent linguistic difficulties affecting functional communication in their everyday life without an apparent reason, are considered to have developmental language disorder, DLD (Bishop et al., 2017). The term DLD has been suggested to replace earlier terms, such as specific language impairment (SLI), language impairment, language disorder, and primary spoken language disorder. DLD manifests as difficulties in expressive language or in both expressive and receptive language. In the present review, the term ‘receptive language’ is used as a synonym to ‘oral language comprehension’, thus excluding reading comprehension. Difficulties in oral language comprehension are known to be persistent and to respond to intervention less well than difficulties in expressive language (Clark et al., 2007; Roberts & Kaiser, 2012). There are indications that problems predominantly in expressive or in receptive language skills are not different in quality, but rather in quantity (Saar et al., 2018). That is, the more severe the disorder, the more oral language comprehension is affected.

Research on oral language comprehension interventions is scarce (Boyle et al., 2010), and intervention research on school-age children and adolescents with receptive difficulties is particularly scarce (Ebbels et al., 2017). More knowledge is needed for these specific age groups as the prognosis of language difficulties changes with age. In young children, some language difficulties are ameliorated through maturation and intervention (Law et al., 2000). If children aged 5 years and older still present language difficulties, it is likely that these difficulties will persist in some form also later in life (Bishop et al., 2017). In addition, the role of language in social communication and learning becomes more substantial the older the children get as the demands on language skills change and grow. The age of the individual may also affect what kind of interventions should be used. Although there is little research conducted on the approach used with children of various ages, it has been suggested that implicit therapy techniques seem to be preferred in young children, whereas techniques using explicit intervention methods may be more appropriate in school-age children and adolescents (Ebbels, 2014). Implicit therapy techniques refer to interventions exposing the child to optimal language, whereas explicit therapy techniques refer to techniques where learning and rules are made explicit for the child. This difference in the therapy techniques used indicates that the interventions intended for school-age children and adolescents may differ from those meant for younger children, and a better understanding is needed of the interventions employed for these specific groups.

The long-lasting linguistic difficulties related to DLD are associated with a high incidence of dyslexia (Catts et al., 2002). School attainment of individuals with language disorder is often lower than in the general population, as is the socio-economic status later in life (Elbro et al., 2011). Linguistic difficulties affect not only academic achievements, but also social relations. Adolescents with DLD have fewer close friendships and poorer quality of friendships than their typically developing peers (Clegg et al., 2005; Durkin & Conti-Ramsden, 2007). Permanent linguistic difficulties are also associated with other risk factors affecting different areas of life: compared to the general population, young adults with DLD are more likely to live at home with their parents, their incidence of unemployment is higher, and they have an increased risk of psychiatric disorders in adult life (Arkkila et al., 2008; Clegg et al., 2005; Elbro et al., 2011). Further, 66–90% of juvenile offenders have below average language skills (Bryan et al., 2007) indicating that functioning in society without adequate linguistic skills is challenging. It should be noted, however, that there is a high comorbidity in DLD with other neurodevelopmental disorders and symptoms which affect performance. It is therefore hard to differentiate the effects caused by linguistic difficulties alone when other symptoms are often also present. It does seem though, that severe linguistic difficulties may be a factor that increases the risk for marginalisation in society. Thus, persistent linguistic difficulties affect not only the individuals, but also society. For example, in Great Britain the costs of marginalisation for one individual have been calculated to be as high as over two million pounds (Coles et al., 2010). The existing risks associated with persistent linguistic difficulties further emphasise the need for interventions for school-age children and adolescents.

### Focus of intervention

Oral language comprehension consists of several different skills and processes from perception, to sufficient working memory, to understanding the meaning of words and structures and how to use them (Morgan, 2013). Therefore, interventions aiming to improve oral language comprehension can target different areas. In the present review the term ‘focus of intervention’ is used to describe the area of language, skill, or process that is targeted in an intervention. The following features have been named as possible foci of oral language comprehension intervention in reviews touching on the topic of oral language comprehension interventions in school-age children and adolescents: receptive vocabulary, semantics, receptive grammar (syntax, morphology), narratives, both expressive and receptive language together, auditory processing and language processing (Boyle et al., 2010; Cirrin & Gillam, 2008; Ebbels, 2014; Law et al., 2003, 2004). These foci of intervention target either language skills or language processing of the participants. Language skills refer to different components of language that are targeted in an intervention, for example, vocabulary, syntax, morphology, or narratives. Targeting language skills seems to be the most common intervention focus in speech and language therapy and there is evidence regarding its efficacy (Cirrin & Gillam, 2008; Law et al., 2003, 2004). Language processing refers to skills or processes that affect not only an area of language but more general language processing skills. Interventions on language processing can target, for example, auditory processing in order to improve language skills (Cirrin & Gillam, 2008). If language processing could be improved, language skills in many domains could possibly be improved simultaneously. Targeting language processing has been conducted by computerised interventions which could potentially save costs. The possibility of both enhancing language skills in many domains and saving costs has probably added to the attractiveness of targeting language processing. However, for now, there is little evidence on the efficacy of targeting language processing to enhance language skills or oral language comprehension (Melby-Lervåg & Hulme, 2013; Strong et al., 2011).

Reviews that touch on the topic of oral language comprehension interventions in school-age children and adolescents (Boyle et al., 2010; Cirrin & Gillam, 2008; Ebbels, 2014; Law et al., 2003, 2004) do not focus solely on school-age children and adolescents with difficulties in comprehending spoken language. To our knowledge, there are no studies examining the possible foci of intervention in this group. It is thus unknown whether all the possible intervention foci have been identified. For example, in children 8 years-of-age and younger, modifying the communicative environment is a common focus of intervention when targeting oral language comprehension (Tarvainen, Stolt & Launonen, 2020). In the reviews touching on the topic of oral language comprehension interventions in school-age children and adolescents, modifying the communicative environment was not mentioned. It is thus unknown whether modifying the communicative environment is a significant focus of intervention in school-age children and adolescents with DLD. Knowledge regarding the focus of intervention is important in order to gain an overview of oral language comprehension interventions, and to be able to provide the best possible interventions for each individual according to their difficulties. At present, it is also unknown whether interventions targeting a specific area of oral language comprehension indicate more or less efficacy than others in school-age children and adolescents with DLD.

### Efficacy of oral language comprehension interventions in school-age children and adolescents

Oral language comprehension interventions with clear efficacy are needed in order to ameliorate linguistic difficulties and to improve the future prospects of individuals with DLD. In the present review, ‘efficacy’ refers to the ability to produce desired results and ‘effect size’ expresses the magnitude of efficacy. There are individual studies suggesting that speech and language therapy interventions for school-age children and adolescents indicate efficacy (see for example Ebbels et al., 2017; Wright et al., 1993). However, there are no reviews focusing solely on oral language comprehension interventions and their efficacy in school-age children and adolescents with DLD. Information on the efficacy of oral language comprehension interventions in this age group has to be collected from reviews focusing on oral language interventions in general (Cirrin & Gillam, 2008; Law et al., 2003, 2004), oral language comprehension interventions in a wide (2–16 years) age scale (Boyle et al., 2010), or from reviews focusing on a specific aspect of language, such as grammar (Ebbels, 2014). Results on the efficacy of oral language comprehension interventions are mixed: a meta-analysis stated that there is no effect (Law et al., 2003, 2004) while more recent reviews reported interventions that had shown a positive effect on oral language comprehension, some with a large effect size (Boyle et al., 2010; Cirrin & Gillam, 2008; Ebbels, 2014). Further, there are no studies on the efficacy of oral language comprehension interventions summarizing recent research. Better knowledge regarding intervention efficacy is needed to provide the best possible interventions, maximise outcomes, and to ameliorate the risks associated with difficulties in oral language comprehension.

### Level of evidence

Intervention studies can be categorised by the level of evidence, i.e. the quality of the evidence. The quality of evidence refers to ‘the methods used by the investigators during the study to minimise bias and control confounding within a study’ (National Health and Medical Research Council (Australia), 2000, p. 14). Knowledge regarding the level of evidence is needed to understand how much confidence one can have in the results of a given study. One example of the level of evidence is the categorisation by the National Health and Medical Research Council, NHMRC (2000). Systematic reviews of randomised controlled trials, RCTs, represent the highest level of evidence, whereas studies with pre-test/post-test design without experimental control represent the lowest level of evidence. There is a great variation in the level of evidence in reviews that touch on the topic of oral language comprehension interventions of school-age children and adolescents. The systematic review of Law et al. (2003, 2004) on speech and language therapy interventions in general included only RCTs, thus presenting a very high level of evidence. In the systematic review of Cirrin and Gillam (2008) examining language intervention practices for school-age children, the level of evidence was evaluated by critical appraisal points. The authors stated that one can have moderate confidence in the results of the included studies with few exceptions. In the only study examining interventions for children and adolescents with receptive-expressive language impairment (Boyle et al., 2010), the studies were classified either as RCTs or phase I and small-scale trials. The level of evidence was not evaluated further. The review of Ebbels (2014), on effectiveness of intervention for grammar, reported whether there was a control group or not, and a description of it when there was one, but no other references related to factors contributing to the level of evidence were made. There is thus no systematic reporting of the level of evidence throughout the reviews touching on the topic of oral language comprehension interventions in school-age children and adolescents. Therefore, the information on the level of evidence, and accordingly the confidence one can have on the results of oral language comprehension interventions, is incomplete and unclear. The information on the level of evidence, however, is crucial to evaluate how much confidence one can have in the results of the intervention in question, and to be able to choose therapy techniques with the most robust knowledge regarding their efficacy.

### Aim of the study

The present review focused on oral language comprehension interventions in school-age children and adolescents with DLD, and is the continuation of a previous review on interventions for 1–8 year-old children with language disorders or difficulties (Tarvainen et al., 2020). The interventions for children aged 8 years and younger focused on the following areas: modifying the communicative environment of the child, targeting aspects of the child’s language, or targeting the child’s language processing. The review suggested that the majority of oral language comprehension interventions indicate efficacy and that researchers and clinicians can have moderate confidence in the results of the included studies, with few exceptions. The present review focuses on the same topic areas, but in school-age children and adolescents. Knowledge regarding the interventions for this specific age group is important, as the information on focus of intervention, efficacy, and confidence in the results gained from level of evidence in school-age children and adolescents is obscure. Similarly, interventions for younger and older children are likely to differ (Ebbels, 2014). Research on the matter is needed to maximise the outcomes of interventions and to enhance individual options in life. The aim of the present review was to identify interventions targeting oral language comprehension in school-age children and adolescents with DLD. Further, the goal was to examine the focus of intervention, efficacy, and level of evidence in this group.

## Methods

### Study design of the present review

A preliminary literature search on oral language comprehension interventions indicated a limited number of studies in general, and a very small number of RCTs. Because of the limited amount of research conducted on the topic, a systematic review including only RCTs was not considered to be the best option, and it was decided to look for evidence from studies conducted with various research designs. The aim was also to develop a qualitative overview on the topic and to summarise the findings of current research. Therefore, a systematic scoping review was chosen as the study design for both the present and the previous review (Tarvainen et al., 2020). Systematic scoping review is a useful method for examining a subject which has little research conducted on it or a broad scope (Arksey & O’Malley, 2005; Armstrong et al., 2011), and it was considered beneficial in conducting a descriptive article on this sparsely researched topic. Further, as there seemed to be very little research on the topic, a relatively large age group was considered adequate to gain an overview on oral language comprehension interventions in school-age children and adolescents. Scoping reviews include five key phases: 1) identifying the research question; 2) identifying relevant studies; 3) study selection; 4) charting the data; and 5) collating, summarizing, and reporting the results (Armstrong et al., 2011). Systematic scoping review protocol has been used in the field of speech and language therapy as a useful method to summarise present knowledge (see for example Smith et al., 2017). The present review systematically followed the five-step scoping review protocol as described by Armstrong et al. (2011) in creating an overview of oral language comprehension interventions.

### Identifying the research question

The research question was created using the PICO framework (Schardt et al., 2007), where P refers to population, I to intervention, C to comparison treatment, and O to outcomes. In this review, the target population was defined as school-age children and adolescents with DLD. The intervention was defined as an intervention aiming to improve oral language comprehension on its own or together with expressive language. No comparison treatment was chosen as the aim was to gain an overview of oral language comprehension interventions and choosing one would have limited the included interventions. The outcomes were skills in one or more areas contributing to oral language comprehension. The research questions were:

1. Which interventions target oral language comprehension in school-age children and adolescents with DLD?
2. What is the focus of intervention in these studies?
3. What is the efficacy of the interventions?
4. What is the level of evidence of the intervention studies?

### Identification of relevant studies

The initial search for this scoping review was carried out in October and November 2016. An update search was conducted in January 2019. After this, the searches were kept up to date by alerts from the databases until the end of August 2020. Studies were identified from the following sources: EBSCOhost, ERIC, LLBA, Ovid, PsycINFO, PubMed, Scopus, and Web of Science. The following search terms were used to identify articles:

Intervention OR rehabilitation OR therapy OR treatment OR training OR enhanc* OR improv*

AND comprehen* OR receptive

AND language impairment* OR language disorder* OR language difficult*

AND child* OR adolesc* OR preschool OR school

NOT aphasi* OR autism.

The present review is the second part of a larger search. The previous review article (Tarvainen et al., 2020) included children aged eight years and younger. The present review focused on children and adolescents aged 9 to 17 years. Some of the studies identified in the search included children younger than eight and older than nine. To include all studies matching the inclusion criteria in either of the two reviews, in the present review there are also studies with participants under the age of nine — the youngest participant is 5;10 (years;months). Therefore, the inclusion criteria regarding the age of the participants in the present review (5 to 17 years) overlaps somewhat with the first review (8 years and younger). The inclusion criteria of the studies are presented in Table 1.

**Table 1.** Inclusion criteria of the studies included.

Participants were 5–17 years old
Participants had developmental language disorder
Participant’s language difficulties manifested in receptive language or in both receptive and expressive language
Study examined the effects of an intervention targeting oral language comprehension independently or along with expressive language
Study was an intervention study reporting original results or a systematic review with or without a meta-analysis
Study had a detailed description of the intervention method used (except systematic reviews containing several methods)
Systematic reviews summarised the results on oral language comprehension
Study had at least one assessment measure examining oral language comprehension before and after the intervention
Study was published in a peer reviewed journal
Study was published in 1996 or later
Study was published in English

One of the aims of the present review was to evaluate the level of evidence of different intervention methods. Systematic reviews were considered to provide significant information on the level of evidence in different intervention methods. Therefore, systematic reviews were included in the present review. To be included, however, the systematic reviews had to summarise the results on oral language comprehension. When the results on oral language comprehension were not summarised, the individual articles included in the systematic reviews were read and included in the present review if they matched the inclusion criteria. The included systematic reviews and meta-analyses were not expected to have a detailed description of the included intervention methods. The intervention description was considered adequate if it was detailed enough to be categorised by focus of intervention.

### Study selection

**Figure 1.**
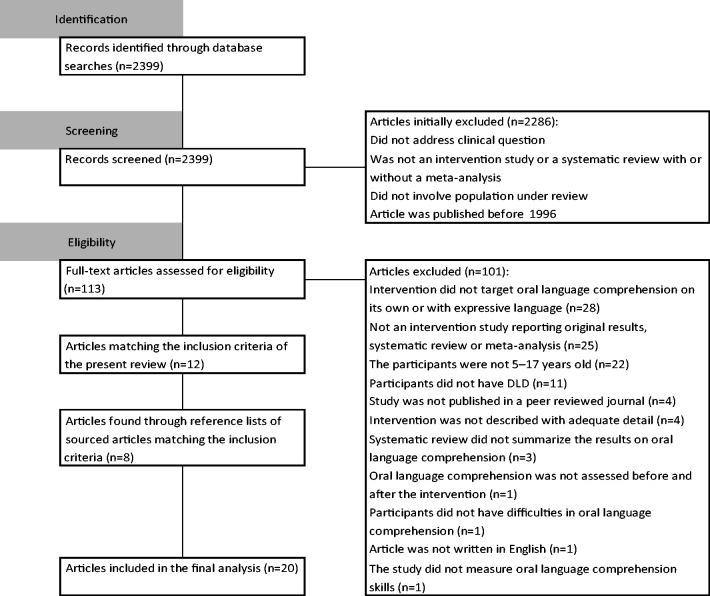
CONSORT flowchart: Identification of articles in the present review.

A total of 2399 citations were found in the database searches. The titles and abstracts were read and, based on this screening, 113 articles were considered relevant. They were chosen for further inspection, and the full text articles were obtained. Based on the full text, 12 articles matched the inclusion criteria. References of systematic reviews found through the database searches and of intervention articles included in the present review were used to search for further articles. A further 8 articles matching the inclusion criteria were identified. The total number of articles included in this review was 20. The 20 articles included 21 studies. Identification of the articles for the present review is presented with a CONSORT flow chart in Figure 1. For simplicity, the results of the initial search, update search, and alerts are treated as one in the CONSORT flowchart.

### Charting the data

The data were charted using Excel software and the following information from the studies was extracted: authors, year of publication, title of the article, participants’ age, diagnoses, number of participants in experimental group and control group, therapy techniques, provider, total intervention hours, duration, mention of bias, results considering oral language comprehension, generalisation, maintenance, intervention focus, effect size, and level of evidence. The total intervention hours were not always stated in the articles. In these cases, they were calculated based on the information in the articles. If this was not possible, the authors were contacted.

### Collating, summarizing and reporting the results

In the present review, only results that considered oral language comprehension were reported, although some of the studies also targeted expressive language (please see Tables 3 [Table table4-23969415211010423]to 5). For example, Balthazar and Scott (2018) examined the use and understanding of complex sentences, but only the results regarding comprehension are discussed in the present review. Focusing only on oral language comprehension possibly leads to a difference between what is reported in the present review and the individual studies included in the present review. For example, the abstract by Joffe et al. (2019) indicates that there were significant improvements in narrative, but not in vocabulary. In the present review the results extracted from the article of Joffe et al. (2019) indicate that there was an improvement in receptive vocabulary in one outcome measure. Narrative comprehension was not assessed individually. It should be noted that some of the included interventions had positive effects on expressive language, but again, those results were not reported in the present review. In addition to the term ‘school-age children and adolescents’, the term ‘children’ is used for simplicity to refer to the participants of the included studies in reporting the results.

#### Focus of intervention

The classification of intervention focus arose from the studies identified in the search. Classification was done based on the criteria used in the present study and may thus differ from that of the original articles. The categorisation of the focus of intervention was based on where the change was expected to happen: whether it was in the child’s skills or processes, or in the communicative environment. There was some overlap between these intervention foci. The intervention foci have been categorised based on what was the most characteristic for the intervention in question. If the aim of an intervention was not explicitly stated in the included article, the study was categorised based on the description of the intervention, and on the outcomes measured. Three different foci were identified in the studies intended for school-age children and adolescents with DLD: 1) targeting aspects of language; 2) targeting language processing; and 3) modifying the communicative environment. The first two foci of intervention targeted the skills of the individual and the last one focused on the individual’s surroundings.

Interventions were categorised as *targeting aspects of language* when the aim was to improve one or more areas of language that affect oral language comprehension. The areas identified were receptive vocabulary, receptive grammar, and comprehension of narratives. The change was expected to be seen in one or more of these language areas affecting oral language comprehension.

Interventions were categorised as *targeting language processing* when they did not target language, but aimed at improving more general language processing skills. The aim was thus not directly to improve one of the language areas affecting oral language comprehension. These interventions targeted aspects like automatisation of skills or improving auditory temporal processing. Metacognitive strategies to help compensating for current language processing skills, such as narrow verbal working memory, were also included in this category. Visualisation, such as Mental imagery, was interpreted to reduce the burden on verbal working memory by transferring verbal information into visual form and was thus categorised as targeting language processing.

Interventions were categorised as *modifying the communicative environment* when the skills of the individual were not directly targeted, but the environment was modified to support the child better. Interventions had to target the communication strategies of the people in the child’s surroundings, such as teachers, to be included in this category. The effect of the modified communicative environment on the individuals’ skills was then examined.

#### Efficacy

The efficacy of interventions was reported via effect size. The effect sizes in the included studies were calculated with Cohen’s d (d), eta squared (η^2^), or partial eta squared (ηр^2^). The categorisation used in the present review follows the values reported in the literature. In Cohen’s d, the minimum values of categories are: very large effect size is 1.2 or higher, large 0.8, medium 0.5, and small 0.2 (Cohen, 1988; Sawilowsky, 2009). In eta squared and partial eta squared, large effect has a value of 0.14 or higher, medium 0.06, and small 0.01. In some of the articles, effect size was not calculated, but researchers reported statistically significant improvement in comprehension skills. In these cases, the efficacy was considered to be ‘statistically significant benefits reported’. In some of the studies the results were mixed: some participants improved in their skills whereas others did not. These mixed results were evident in only time series design and pretest/post-test–design where individual patterns of improvement were examined. In group-level studies, the possible variation in the results cannot be detected. In the interventions which indicated to have no effect on oral language comprehension skills, efficacy was considered to be ‘no effect’.

#### Level of evidence

The studies were categorised by the level of evidence according to the classification of the NHMRC into six categories (NHMRC, Australia, 2000). This categorisation was chosen as it has been developed in multidisciplinary committees with a rigorous evidence-based approach and is well-known. The levels of evidence and the categorisation criteria are presented in Table 2.

**Table 2.** Designation of levels of evidence according to the National Health and Medical Research Council.

Level of evidence	Study design
I	Evidence obtained from a systematic review of all relevant randomised controlled trials.
II	Evidence obtained from at least one properly-designed randomised controlled trial.
III-1	Evidence obtained from well-designed pseudorandomised controlled trials (alternate allocation or some other method).
III-2	Evidence obtained from comparative studies (including systematic reviews of such studies) with concurrent controls and allocation not randomised, cohort studies, case-control studies, or interrupted time series with a control group.
III-3	Evidence obtained from comparative studies with historical control, two or more single arm studies, or interrupted time series without a parallel control group.
IV	Evidence obtained from case series, either post-test or pretest/post-test.

**Table 3.** Intervention studies: Targeting aspects of language.

Study	Focus of intervention	Level of evidence	N	Control group	Age, years	Diagnosis	Therapy techniques	Total hours	Outcome measures	Results	Efficacy	Maintenance	Generalisation
Parsons et al., 2005	Vocabulary	IV	2	no	8–9	SLI	Becoming a Word Wizard: semantic-phonologic approach of teaching curriculum-based words	9	British Picture Vocabulary Scale (BPVS); Researcher-created word comprehension task	No change on BPVS. Understanding of words improved in researcher-created assessment. Error styles changed: no more phonologic or unrelated errors, only semantic errors.	Effect size n/a, statis-tically significant benefits reported	n/a	Understanding of control words improved. The improvement was statistically significant.
Wright et al., 2018	Vocabulary*	IV	25	no	9–16	DLD or LD+ASD	Semantic-phonologic approach	3.25	Researcher-created tasks: Lexical decision (LD) (real word or not); multiple choice (MC) (which of 3 definitions match the word); self-evaluation of word knowledge	In all three tasks knowledge of target words improved more than control words	Medium (d=0.74 self-evaluation of word knowledge; n/a for lexical decision or multiple choice). (A very large effect size (ηp2=0.63) was detected when LD, MC & two production tasks were assessed.)	n/a	Treatment lead to greater generalisation in the case of verbs than nouns.
Lowe & Joffe, 2017	Vocabulary*	IV	15	no	13–14	LD	Phonological-semantic approach	7.5	Researcher-created word knowledge task where students evaluate their own knowledge of words	Knowledge on experimental and control words improved. In low frequency words the improvement in experimental words was better than in control words.	Effect size n/a, statistically significant benefits reported	After 7 weeks the results remained	n/a
Ebbels et al., 2014	Grammar, comprehension of coordinating conjunctions	II	7	7 waiting controls	11–16	Severe RELI. Some had also other difficulties or diagnoses	The SHAPE CODING system, explicit teaching with visual support	4	Researcher-created task on comprehension of conjunctions; blocks on the TROG-2 which test the targeted conjunctions.	Comprehensionof coordinatingconjunctionsimproved	Very large (d=1.33 in combined results of test on comprehension of conjunctions & blocks on the TROG-2 which test the targeted conjunctions.	4 months, results remained	Progress generalised to the overall TROG-2 scores. No generalisation to the designed control structure (comprehending passives).
Ebbels & Lely van der, 2001	Grammar, comprehension of passive and wh-questions	III-3	4	no	11–12	severe SLI	The SHAPE CODING system, explicit teaching with visual support	29	Researcher-created tasks: an acting-out procedure, a picture selection task (Test of Active and Passive Sentences, TAPS) and ‘who’ and which’ questions.	Comprehension of passive an wh-questions improved	Effect size n/a, statis-tically significant benefits reported	10 weeks, results maintained in 3/4 children	n/a
Ebbels, 2007: Study 1	Grammar, comprehen-sion of dative form	III-3	3	no	12–14	severe SLI	The SHAPE CODING system, explicit teaching with visual support	10	Researcher-createddativecomprehensiontask	Comprehension of dative form improved in 2/3 children	Effect size n/a, statistically significant benefits reported in 2/3 children	n/a	n/a
Ebbels, 2007: Study 2	Grammar, comprehen-sion of comparative questions	III-3	2	no	n/a	severe SLI	The SHAPE CODING system, explicit teaching with visual support	10	Researcher-created comparative questions comprehen-sion task	Comprehension of comparative questions improved	Very large & no effect size reported (d=1.98 in one participant, no effect size for other, but statisticallysignificantimprovement)	n/a	n/a
Levy & Fried-mann, 2009	Grammar, syntactic movement*	IV	1	28 td, no int.	12	SLI	Explicit teaching of syntactic movement using visual support	11	BAMBI: sentence-picture matching task; BAFLA: question-picture matching test	Comprehension of wh-questions improved. Comprehension of object relative clauses improved to match age level.	Effect size n/a, statis-tically significant benefits reported	10 months later results remained	Resultsgeneralised to untrained conditions
Zwitserlood et al., 2015	Grammar, relative clauses. Production targeted, but comprehension also of interest.*	III-3	12	no	9–12	SLI	MetaTaal: explicit teaching with visual support	5	Researcher-created relative clause task	No significant effects on oral language comprehension	No effect	12 weeks, no effect	n/a
Balthazar & Scott, 2018	Grammar, understanding and use of complex sentences*	IV	1x/ week 14, 2x/ week 16	no	10–14	SLI	Exposure and repetition, identification & scaffolded manipulation activities	1x/ week 7.5; 2x/ week 15	CELF-4: Concepts and Following Directions (CFD) & CASL: Sentence Comprehension subtest (SC)	Oral language comprehension improved. The test scores improved more in 2x/w than in 1x/w group.	Both groups together: Large & not statis-tically significant (Ƞ²= 0.30 in CFD, the effect of time for SC of the CASL was not statistically significant)	n/a	n/a
Joffe, Rixon & Hulme, 2019	Vocabulary & narratives*	II	narrative 84, vocabulary 82, combined 84	83 waiting controls	12	LD	Narrative: understand-ing and telling of stories, using the story structure. Vocabulary: developing key concepts & vocabulary items relevant to the curriculum	16	BPVS-2; subtests of the TOWK: single-word receptive vocabulary & figurative language; researcher-created receptive vocabulary (RV) task.	Comprehension of words related to the themes in the intervention improved in vocabulary and combined group.	Small & no effect (d=0.27 in vocabulary group & 0.34 in combined group in the researcher-created RV task; no effect in narrative group). No effect on BPVS-2 or TOWK in any group.	n/a	There were improvements across a range of stimuli that were not targeted in the intervention suggesting generalised learning
Petersen et al., 2008	Narratives. Expression of more interest, comprehension also measured.*	IV	12	no	6–9	LI	Narrative-based language intervention	24	Test of Narrative Language: Narrative comprehension composite score (NC)	Oral language comprehension improved	Large (d=0.81 in NC)	n/a	n/a

Note. SLI = Specific Language Impairment; n/a = Information not available; * = Both oral language comprehension and expressive language targeted; LD = language disorder; ASD = autism spectrum disorder; d = Cohens’s d; ηp2 = partial eta squared; RELI = Receptive and expressive language impairment; TROG-2 = Test of Reception of Grammar; CELF-R = Clinical Evaluation of Language Fundamentals; BPVS-II = British Picture Vocabulary Scales; TROG = Test of Reception of Grammar; td = typically developing children; no int. = no intervention; BAMBI = Battery for assessment of syntactic abilities in children; BAFLA – Friedmann’s battery for agrammatism; CELF-4 = the Clinical Evaluation of Language Fundamentals–Fourth Edition; CASL = Comprehensive Assessment of Spoken Language; ƞ2 = eta squared; BPVS-2 = British Picture Vocabulary Scale, 2nd edition; TOWK = Test of Word Knowledge; LI = Language impairment.

In the classification of NHMRC, the level of evidence of a systematic review corresponds to the level of evidence of the included studies (NHMRC, Australia, 2000). Systematic reviews, including articles of various study designs, were therefore categorised based on the lowest level of evidence of the included articles also in the present study. Properly designed randomised controlled trials at level II were defined as RCTs with random allocation (cluster randomisation included), blinded assessors after the intervention, and reported attrition. Cluster randomisation had to include more than two groups to be categorised as random allocation. One study (Starling et al., 2012) was considered to be at level III-1 even though the title of the article suggests the study design to be a RCT. The decision was made because there were only two schools which were randomly allocated. The effect of the school was not considered to be eliminated by the random allocation including only two schools. Well-designed pseudorandomised trials at level III-1 were defined as trials with blinded assessors after the intervention and reported attrition. If the study lacked the required characteristics, it was designated to a one-level–lower category. Studies without a control group which had two or more intervention groups that were not compared with each other were considered to be single arm studies. They were designated to level III-3. All studies using time-series design without a control group were designated to level III-3. Studies were categorised as pretest/post-test design also when the measures for oral language comprehension were administered in this design even though the general design of the study was time series design, like in the case of Balthazar and Scott (2018). Studies with only one intervention group and no control group were categorised as pretest/post-test designs to level IV. No studies with only post-test measures were included as they failed to match the inclusion criteria (see Table 1).

In the present review, studies on level I were considered to provide a very high level of confidence considering the results. Level II studies were considered to provide a high level of confidence, whereas level III-1 to III-3 were considered to provide moderate confidence in the results. Level IV studies were considered to provide only indicative confidence in the results as they lack experimental control.

#### Reliability

A researcher blind to the results of the present review categorised independently randomly selected 33% (7/21) of the studies on the focus of intervention and the level of evidence. The agreement of the categorisations made by the researcher and the first author were calculated. The agreement between the two independent categorisations were as follows: focus of intervention 86% (6/7) and level of evidence 86%. A consensus on the categorisation was reached after discussion.

## Results

### Description of the studies

The 21 studies included in the present review examined the efficacy of a specific intervention method or methods, or compared two or more intervention methods to each other (see Tables 3 [Table table4-23969415211010423]to 5). One systematic review was identified that matched the inclusion criteria (Fey et al., 2011). Of the 21 studies, 12 targeted both oral language comprehension and expressive language together. The other nine intervention studies aimed solely at improving oral language comprehension. Altogether 1661 children aged 5;10–16;1 (years;months) participated in these 21 studies. The diagnoses of the participants were: specific language impairment (SLI; 10 studies); language impairment, (LI; 7 studies); language disorder (2 studies); auditory processing disorder and/or primary spoken language disorder (1 study); and DLD or language disorder with autism spectrum disorders (1 study). All of the diagnoses stated above were considered to fall under the term DLD, except language disorder with autism spectrum disorder. The study in question (Wright et al., 2018), with seven participants diagnosed with language disorder with autism spectrum disorder was included as most of the participants (18 of 25) had a diagnosis of DLD.

Maintenance was reported in 38% (8/21) of the studies. The maintenance phase varied between 7 weeks and 10 months. In all of the studies reporting efficacy on oral language comprehension in which the maintenance was evaluated, the results remained after the maintenance phase, except in the study of Ebbels et al. (2001) where the results were maintained only in three of the four participants. Generalisation was reported in 33% (7/21) of the studies. When generalisation was reported in the studies indicating efficacy, it suggested some generalisation also to untrained conditions.

### Focus of intervention

#### Targeting aspects of language

Intervention studies targeting aspects of language (12/21, 57%) focused either on receptive vocabulary, comprehension of grammar (morphology and/or syntax), comprehension of narratives, or receptive vocabulary and comprehension of narratives together (Table 3). The interventions were provided by a (speech and language therapist, SLT) researcher, the child’s usual SLT, a teaching assistant, or an SLT student.

**Table 4. table4-23969415211010423:** Intervention studies: Targeting language processing.

Study	Focus of intervention	Level of evidence	N	Control group	Age, years	Diagnosis	Therapy techniques	Total hours	Outcome measures	Results	Efficacy	Maintenance	Generalisation
Fey et al., 2011	Auditory temporal processing*	IV, syste- matic review	Total number of participants in 27 studies 677		6–12	APD and/or primary spoken language disorder	Auditory interventions including ‘traditional listening’ treatments, AIT, Fast ForWord & Earobics	3–60, mean 32	Several	No effects on oral language comprehension	No compelling evidence on the efficacy of auditory interventions	n/a	n/a
Gillam et al., 2008	Auditory temporal processing*	II	54	CALI 54, ILI 54, AE 54	6–9	LI	Fast ForWord (FFW), computerised training with modified acoustic signals	50	Token Test for Children	No significant difference between the groups, all groups improved in their oral language comprehension skills.	No difference between the groups.	3 months & 6 months, improvements in all groups. Small to medium (d= CALI 0.54, FFW 0.66, ILI 0.56, AE 0.35 after 6 months on the Token Test).	n/a
Cohen et al., 2005	Auditory temporal processing*	II	23	Computer based activities+ regular SLT 27, regular SLT 27	6–10	SLI	FFW, computerised training with modified acoustic signals + regular SLT	45	CELF-3, TOLD-P	No significant difference between the groups, all groups improved in their oral language comprehension skills.	No additional effect by FFW or computer-based activities.	6 months, all groups made gains during maintenance	n/a
Friel-Patti et al., 2001	Auditory temporal processing*	IV	5	no	5–9	LI	FFW, computerised training with modified acoustic signals	47	Token Test for Children	No effect in four participants on oral language comprehension, improvement in one participant	Effect size n/a, mixed results	n/a	n/a
Bishop et al., 2006	Auditory temporal processing & automatisation	III-1	Modified speech 12, slow speech 12	9 no int.	8–13	Receptive LI	computerised training to train grammatical comprehension	2–7	TROG-2 & ERRNI (comprehension scale)	No effects on oral language comprehension	No effect	n/a	n/a
Hsu & Bishop, 2014	Automatisation	III-1	28	20 + 48 td	6–11	SLI	Computerised training of two prepositions	0.5	TROG-E	No effects on oral language comprehen- sion	No effect	n/a	Improvement in the training did not generalise to general oral language comprehension
Joffe, Cain & Marić, 2007	Reducing the burden on verbal working memory by using visualisation	IV	9	16 td	9	SLI	Teaching children to produce mental images to help to understand and remember sentences	2.5	Story comprehension task by Bishop & Adams	Story comprehension improved as measured by the ability to answer questions about it	Large (ηр²=0.608 in answering literal questions)	n/a	n/a
Dixon et al., 2001	Reducing the burden on verbal working memory by using visualisation	III-3	8	no	9–15	LI	Visualising & verbalising (V&V) and/or ‘traditional therapy’	5	Researcher-created task: Analytical Reading Inventory where the sections where read out loud to the participants	No difference between V&V and traditional therapy. Oral language comprehension improved regardless of therapy method.	Effects size n/a, statis-tically significant benefits reported	n/a	n/a

Note. * = The intervention focused both on oral language comprehension & expressive language; APD = Auditory processing disorder; AIT = auditory integration training; n/a = Information not available; CALI = Computer-assisted language intervention; ILI = individualized language intervention; AE = Academic enrichment; LI = language impairment; SLT = Speech and language therapy; SLI = Specific language impaiment; CELF-3 = Clinical Evaluation of Language Fundamentals—Third Edition UK; TOLD-P = Test of Language Development—Primary; OWLS = Oral and Written Language Scales; no int. = no intervention; TROG-2 = Test for Reception of Grammar; ERRNI = Expression, Reception and Recall of Narrative Instrument; td = Typically developing children; TROG-E = Test for Reception of Grammar-Electronic; ηр² = partial eta squared.

**Table 5.** Intervention studies: Modifying the communicative environment of the child.

Study	Focus of intervention	Level of evidence	N	Control group	Age, years	Diagnosis	Therapy techniques	Total hours	Outcome measures	Results	Efficacy	Maintenance	Generalisation
Starling et al., (2012)	Modification of teacher's language*	III-1	21	22 waiting controls	12–14	LI	Modification of written & oral language, paying attention to information processing, direct vocabulary instruction	8	WIAT–II: Listening Comprehension	Oral language comprehension improved	Medium (ηр²= 0.106 WIAT–II: Listening Compre- hension)	12 weeks, average standard scores remained stable	Children enjoyed tasks more, became more engaged & developed improved listening comprehension abilities

Note. * = Both oral language comprehension and expressive language targeted; LI = Language impairment; WIAT–II = Wechsler Individual Achievement Test—Second Edition; ηр² = partial eta squared

Three studies targeted receptive vocabulary (Lowe & Joffe, 2017; Parsons et al., 2005; Wright et al., 2018). They all used some variation of semantic-phonologic approach to improve receptive vocabulary. Semantic-phonologic approach refers to a therapy technique where both the meaning (semantics) and the phonological form of the word are discussed and worked with. The age of the participants in these studies varied between 8–16 years. All studies reported benefits relating to the participant’s receptive vocabulary skills. In the study by Wright et al. (2018), participants’ self-reports on word knowledge were also examined. The self-reports indicated growth in word knowledge with a medium effect size. In one study, receptive vocabulary was targeted together with narratives in 12-year-old children (Joffe et al., 2019). Developing key concepts and vocabulary items relevant to the curriculum resulted in improvement in receptive vocabulary on one measure with a small effect size.

Seven studies targeted receptive grammar. Participants in these studies were 9–16-year-old children. Six of the intervention studies used explicit teaching of the grammatical rules with visual support: the SHAPE CODING system (Ebbels, 2007; Ebbels et al., 2014; Ebbels & Lely van der, 2001), MetaTaal (Zwitserlood et al., 2015), and explicit teaching of syntactic movement (Levy & Friedmann, 2009) were used. In the seventh study, exposure, repetition, identification, and scaffolded manipulation activities were used (Balthazar & Scott, 2018). Here scaffolding means a cue, a prompt, or an explanation. Of these seven studies, six indicated efficacy in improving participants’ grammar comprehension ability—some with a very large effect size. The only study not reporting improvement in oral language comprehension was that using MetaTaal technique (Zwitserlood et al., 2015).

The only study which focused solely on comprehension of narratives used a therapy technique called Narrative-based language intervention, NBLI (Petersen et al., 2008). In NBLI, children are taught the typical elements of a story, the so called ‘story grammar’. Knowledge of story grammar was considered to help comprehending narratives. The use of NBLI in the study of Petersen et al. (2008) had a large effect on oral language comprehension in children aged 6–9 years.

#### Targeting language processing

Intervention studies categorised as aiming to *improve language processing* (8/21, 38%) targeted auditory temporal processing, automatisation of specific skills, or reducing the burden on verbal working memory (Table 4). The interventions were provided by (SLT) researchers. Computerised training was used in targeting auditory temporal processing and automatisation. This training was supervised by school staff, parents, clinicians, or graduate students in speech and language therapy.

Auditory temporal processing was targeted in five studies using computerised training with acoustically modified speech. The interventions used different auditory interventions, including the Fast ForWord Language program. Participants in these studies were 6–13-year-old children. Three of the five studies, including one systematic review, found no effect on oral language comprehension using acoustically modified speech (Bishop et al., 2006; Cohen et al., 2005; Fey et al., 2011). One of the five studies compared acoustically modified speech (Fast ForWord) with computer-assisted language intervention, individualised language intervention and academic enrichment in 6–9-year-old children (Gillam et al., 2008). No significant difference was found between the four groups. One study found mixed effects indicating that one of the five 5–9-year-old participants seemed to benefit from the intervention, whereas four others did not (Friel-Patti et al., 2001).

Two studies aimed to automatise specific skills (Bishop et al., 2006; Hsu & Bishop, 2014). One (Bishop et al., 2006) focused also on auditory processing using modified speech. Practice was done with a computer program where the 6–13-year-old participants executed repetitive tasks in order to learn a small set of words. Neither of the studies found a positive effect on oral language comprehension skills.

Two studies were categorised as using compensatory metacognitive strategies to support current processing skills (Dixon et al., 2001; Joffe et al., 2007). They both used visualisation to reduce the burden on verbal working memory and to compensate for the difficulties narrow verbal working memory would cause. In the study by Joffe et al. (2007) a technique called Mental imagery was used. In Mental imagery the children were taught ‘to think in pictures’ as this would help them to understand and remember discourse better. It was found to have a large effect on comprehending literal questions in a story comprehension task in 9-year-old children. Comprehension of inferential questions did not improve even though this was one of the aims of the intervention. In the study by Dixon et al. (2001) a technique called ‘Visualising and verbalising’ was compared with ‘traditional therapy’ in 9–15-year-old children and adolescents. In Visualising and verbalising the aim was to improve mental imagery skills. The children were also asked to verbally describe the mental images. No difference was found between the two groups and the authors reported Visualising and verbalising and traditional therapy to be equally beneficial for oral language comprehension. Despite the results, the authors of the study had a somewhat critical perspective towards Visualising and verbalising, apparently due to the earlier exaggerated claims made regarding its benefits.

#### Modifying the communicative environment

The communicative environment of school-age children was modified in only one study (1/21, 5%) (Table 5). In this study, the teachers’ communication and language (both oral and written language) skills were discussed with a speech and language therapist (Starling et al., 2012). Attention was also given to direct vocabulary instruction and information processing. This study found a medium effect size on the 12–14-year-old pupils’ oral language comprehension.

### Efficacy

The efficacy of the interventions varied between no effect and a very large effect (see Tables 3 [Table table4-23969415211010423]to 5). Of the included studies, 33% (7/21) reported effect sizes from small to very large, indicating that the therapy technique in question had positive effects on oral language comprehension. A very large effect size was found in one study (Ebbels et al., 2014), a large effect size in three studies (Balthazar & Scott, 2018; Joffe et al., 2007; Petersen et al., 2008), a medium effect size in two studies (Starling et al., 2012; Wright et al., 2018), and a small effect size in one study (Joffe et al., 2019). The therapy techniques with the largest effect sizes were The SHAPE CODING system (Ebbels, 2007; Ebbels et al., 2014), narrative-based language intervention (Petersen et al., 2008), mental imagery (Joffe et al., 2007), modification of teachers’ language (Starling et al., 2012), and semantic-phonologic approach (Wright et al., 2018). Of the included studies, 24% (5/21) reported statistically significant benefits but stated no effect size (Dixon et al., 2001; Ebbels & Lely van der, 2001; Levy & Friedmann, 2009; Lowe & Joffe, 2017; Parsons et al., 2005). Altogether 57% (12/21) of the interventions thus indicated positive results in the 5–16-year-old children’s oral language comprehension. Mixed results were seen in 14% (3/21) of the studies, indicating that the skills of some participants, but not all, improved as a result of the intervention (Ebbels, 2007, study 1; Friel-Patti et al., 2001; Joffe et al., 2019). Of the included studies, 29% (6/21) had no effect on oral language comprehension of the participants (Bishop et al., 2006; Cohen et al., 2005; Fey et al., 2011; Gillam et al., 2008; Hsu & Bishop, 2014; Zwitserlood et al., 2015).

The used outcome measures were clinical tests or researcher-created tasks. Some clinical tests were also modified for the purpose of the study, like in the study of Dixon et al. (2001) where the authors used a reading test as a material that was read out loud to the participants. Researcher-created outcome measures were used in 43% (9/21) of the studies. The efficacy of these studies varied between a very large effect and no effect. Seven of the nine studies (78%) indicated a positive effect on the participants’ oral language comprehension. Both researcher-created tasks and clinical tests were used in 14% (3/21) of the studies. Clinical tests were used in 43% (9/21) of the studies. The efficacy of these studies varied between a large effect and no results. Four of these nine studies (44%) indicated efficacy. In the studies included in the present review, the efficacy was thus indicated more often by researcher-created tasks than by clinical tests. Also, the effect sizes detected with researcher-created tasks were larger than in clinical tests.

Efficacy varied in relation to the intervention focus. Interventions *targeting aspects of language* indicated efficacy in 75% (9/12) of the studies. Interventions *targeting language processing* indicated efficacy in 38% (3/8) of the studies. The studies which focused on auditory temporal processing and automatisation were those with the least effect on oral language comprehension. The only systematic review indicated that there is no compelling evidence on the efficacy of auditory interventions (Fey et al., 2011). Interventions which used metacognitive strategies to compensate current processing skills both indicated efficacy. The only study *modifying the communicative environment* indicated efficacy with a medium effect size (Starling et al., 2012).

### Level of evidence

The level of evidence (see Table 2 for designation of level of evidence) in the included studies varied between II and IV. No systematic reviews of RCTs matching the inclusion criteria were identified, and thus, no study reached level I (i.e. very high level of confidence on the results). The only systematic review identified (Fey et al., 2011) included studies with various research designs, including pre-test/post-test design, and it was designated to level IV.

Of the included studies, 19% (4/21) were randomised controlled trials and were designated to the level of evidence II. According to the classification used in the present review they provide high confidence in the results. In these studies, The SHAPE CODING system was used to improve grammar comprehension skills (Ebbels et al., 2014; very large effect size), Fast ForWord was used to improve auditory temporal processing (Cohen et al., 2005; Gillam et al., 2008; not more effective than other conditions or no effect on oral language comprehension), and key concepts were developed to improve receptive vocabulary (Joffe et al., 2019; small effect size on vocabulary).

The level of evidence from III-1 to III-3 is considered to provide a moderate level of confidence in the results. Of the included studies, 38% (8/21) were on these levels of evidence. Three studies were designated to level III-1 (Bishop et al., 2006; Hsu & Bishop, 2014; Starling et al., 2012). Teacher’s language was modified to improve listening comprehension (Starling et al., 2012; medium effect size). The two other studies at this level targeted automatisation (Hsu & Bishop, 2014) or used computerised training to improve auditory temporal processing and automatisation (Bishop et al., 2006). Neither had an effect on oral language comprehension of the participants. None of the studies were designated to level III-2. Five studies were designated to level III-3 (Dixon et al., 2001; Ebbels, 2007, study 1 & 2; Ebbels & Lely van der, 2001; Zwitserlood et al., 2015). The SHAPE CODING system was used to improve receptive grammar (Ebbels, 2007; Ebbels & Lely van der, 2001). The results varied between no effects to very large effect. MetaTaal was used to improve receptive grammar, but no effect on oral language comprehension was detected (Zwitserlood et al., 2015). Visualising and verbalising was reported to aid oral language comprehension, but the effect size was not calculated (Dixon et al., 2001).

Of the included studies, 43% (9/21) used pre-test/post-test design and were designated to level of evidence IV. This was the most common level of evidence in the studies included in the present review. The studies in which following therapy techniques were used provide indicative confidence in the results: Semantic-phonologic approach (Lowe & Joffe, 2017; Parsons et al., 2005; Wright et al., 2018); explicit teaching of syntactic movement using visual support (Levy & Friedmann, 2009); exposure, repetition, identification, and scaffolded manipulation activities (Balthazar & Scott, 2018); narrative-based language intervention (Petersen et al., 2008); visualisation (Joffe et al., 2007); and, Fast ForWord (Friel-Patti et al., 2001). As already mentioned, the systematic review by Fey et al. (2011) was also designated level IV. It should be noted though, that the confidence one can have on the results of the systematic review of Fey et al. (2011) is higher than the individual studies at level IV conducted with pretest/post-test design. The systematic review of Fey et al. (2011) concluded that different auditory interventions aiming to improve auditory temporal processing had no effect on oral language comprehension.

As the level of evidence provides information on the confidence one can have on the results, it is reasonable to examine the level of evidence in relation to the efficacy of different intervention studies. The level II studies, providing high confidence on the results, indicated efficacy in 50%, that is, in two out of four studies (Ebbels et al., 2014; Joffe et al., 2019). The studies providing moderate confidence in the results at level III-1 to III-3 indicated efficacy in 63% (5/8) of the studies (Dixon et al., 2001; Ebbels, 2007 study 1 & 2; Ebbels & Lely van der, 2001; Starling et al., 2012). The level IV studies, considered to provide indicative confidence in the results, indicated efficacy in 78% (7/9) of the studies (Balthazar & Scott, 2018; Joffe et al., 2019; Levy & Friedmann, 2009; Lowe & Joffe, 2017; Parsons et al., 2005; Petersen et al., 2008; Wright et al., 2018). Most of the studies indicating efficacy provide thus moderate or indicative confidence in the results.

## Discussion

The aim of the present review was to identify interventions targeting oral language comprehension in school-age children and adolescents with DLD. The purpose was also to examine the focus of intervention, efficacy, and level of evidence of these interventions. There is little knowledge regarding oral language comprehension interventions in this group even though the risks associated with persistent linguistic difficulties are evident. Twenty-one studies were identified, including 1661 participants aged 5–16-years. Three different foci of intervention were found in the included studies: targeting aspects of language, targeting language processing, and modifying the communicative environment. Of the included studies, 57% reported efficacy in improving oral language comprehension. The level of evidence in the included studies varied between II and IV, the most common being IV. The results suggest that a careful choice of therapy techniques is required when targeting oral language comprehension in school-age children and adolescents.

### Focus of intervention

*Targeting aspects of language* was the most common focus in the present review. In these studies, participants’ receptive vocabulary, receptive grammar or narrative comprehension skills were targeted. These areas, as well as semantics, have been named previously as targets of interventions aiming to improve oral language comprehension in school-age children and adolescents (Boyle et al., 2010; Cirrin & Gillam, 2008; Ebbels, 2014; Law et al., 2003). In the studies included in the present review, targeting semantics was conducted as a part of interventions using semantic-phonologic approach to improve vocabulary. Therefore, in the present study, targeting semantics is seen as a way of improving receptive vocabulary. Further, receptive and expressive language together has been one of the previously mentioned foci of intervention in the individual studies included in one of the previous reviews (Law et al., 2003). Expressive and receptive language have been targeted together also in the studies included in the present review. However, the areas of language where both expressive and receptive language have been targeted have been defined. As there are several possible areas that oral language comprehension interventions can target, merely saying that both expressive and receptive language are targeted is not specific enough.

*Targeting language processing* was the second most common focus of intervention identified in the present study. The targeted areas in the included studies were auditory temporal processing, automatisation, these two together, and reducing the burden on verbal working memory by visualisation. In previous reviews (Boyle et al., 2010; Cirrin & Gillam, 2008) auditory processing and language processing have been named as targeted areas in interventions, whereas automatisation has not. The most recent research no longer seems to target auditory processing, probably as a result of the negative research findings on its efficacy (see for example Strong et al., 2011). The results for attempting to improve automatisation have not been encouraging, either, according to the two studies identified in the present review (Bishop et al., 2006; Hsu & Bishop, 2014). As difficulties related to oral language comprehension are persistent, there is a need for strategies to cope with the difficulties (Boyle et al., 2010). Reducing the burden on verbal working memory by visualisation (Dixon et al., 2001; Joffe et al., 2007) can be seen as a strategy to help children function with their verbal working memory. However, compensating for current language processing skills by using visualisation has not been named as a target in the previous reviews touching on oral language comprehension in school-age children and adolescents. The results of the present review suggest that it might be more reasonable to focus on these compensatory techniques instead of trying to improve language processing skills.

In this review, *modifying the communicative environment* was also used to ease the language problems of school-aged children. It has not previously been named as a focus of intervention in school-age children and adolescents, although it is a common focus of intervention in young children (Roberts et al., 2019; Roberts & Kaiser, 2011; Tarvainen et al., 2020). Modifying the communicative environment can be seen as a way to help the child function better with his or her current skills, not necessarily as a way to improve the skills. In the study by Starling et al. (2012), modifying teachers’ language resulted in improvement in children’s skills detected with a clinical test. This indicates that the 12–14-year-old children not only functioned better in the class with their current skills, but their skills improved when the communicative environment was more supportive. Modifying the communicative environment seems thus to be an efficient way of supporting oral language comprehension in school-age children and adolescents. Still, modifying the communicative environment was the least used intervention focus in the present review: there was only one study with this focus. It should be assessed whether this way of working to improve oral language comprehension could be a more commonly used approach also in school-age children and adolescents.

The present systematic scoping review provided information on the focus of intervention of oral language comprehension interventions in school-age children and adolescents with DLD. The summary of the possible intervention foci provides new and more precise information on what to target when improving oral language comprehension. Examination of the intervention foci also provides information on what still remains to be researched. In comparison to the processes and skills needed for oral language comprehension (Morgan, 2013), it can be concluded that there were no interventions focusing on pragmatics as a way to aid oral language comprehension. Whether this is due to the search parameters, or the fact that there are no studies focusing on this area, remains unclear. An article identified elsewhere targeting idiom identification, interpretation, explanation and use (Benjamin et al., 2020) suggests the former. Further research on targeting pragmatics as a mean to aid comprehension is needed.

The same three foci of intervention were identified in our previous review, which focused on oral language comprehension interventions in children 1–8 years-of-age with language disorders or difficulties (Tarvainen et al., 2020). Still, the interventions differ from each other depending on the age group. Explicit therapy techniques were common in school-age children and adolescents, whereas implicit therapy techniques were more commonly used in children aged 8 and younger. Semantic-phonologic approach, narrative-based language intervention, and explicit teaching of grammar, as in The SHAPE CODING system, are examples of explicit therapy techniques used with school-age children and adolescents. This finding of differences according to the age of the child aligns with the view of Ebbels (2014) that explicit therapy techniques may be appropriate with school-age children and adolescents, whereas implicit techniques may be more effective for younger children. It has to be noted though, that the efficacy of implicit and explicit therapy techniques in different age groups has not been examined, and the difference may simply reflect clinicians’ bias towards using a particular technique with a specific age group without evidence to support the practice. Further, the results of the present review suggest that metacognitive strategies can also be used to support oral language comprehension in school-age children and adolescents (see also Tarvainen et al., 2020). For example, using mental imagery (Center et al., 1999; Joffe et al., 2007) can be seen as a strategy to better function with current, possibly limited, verbal working memory skills. This technique has been used in children aged seven years and older. In a therapy technique called ‘Lexicon Pirate’, strategies have also been used with positive results in children from 4-years-of-age to school-age children (mean age 9 years) to enhance word finding or to improve receptive vocabulary (Motsch & Marks, 2015; Motsch & Ulrich, 2012). This indicates that even quite young children may benefit from metacognitive strategies. The use of metacognitive strategies still requires further research to verify efficacy in different domains of oral language comprehension and in different age groups.

### Efficacy

The objective of oral language comprehension intervention research should be to examine which interventions indicate efficacy in improving oral language comprehension, and what the magnitude of the effect is. The efficacy of oral language comprehension interventions should thus not be examined as one entity as the interventions differ greatly from one another as does their efficacy. Efficacy also seems to differ by the focus of intervention. In the present review, targeting aspects of language and modifying the communicative environment indicated the most efficacy. When the interventions aimed to improve language processing, there was very little evidence of efficacy on oral language comprehension. Compensating for current processing skills, however, indicated efficacy in improving oral language comprehension. These compensatory strategies are important as DLD with difficulties in oral language comprehension is a lifelong condition and learning to function with it is elementary.

Intervention studies *targeting aspects of language* examined the efficacy of receptive vocabulary, comprehension of grammar, and narratives. Receptive vocabulary interventions were found to have positive results on oral language comprehension in school-age children and adolescents. This aligns with a meta-analysis among younger children—vocabulary interventions have been found to have a positive impact on oral language comprehension (Marulis & Neuman, 2010). An individual intervention study targeting vocabulary among 11–14-old adolescents with language disorder also reported positive effects of an intervention using phonological-semantic activities on the students’ word knowledge (Lowe et al., 2019). There also seems to be other ways, than those identified in the searches, to support receptive vocabulary. The therapy technique called ‘Lexicon Pirate’ (Motsch & Marks, 2015) incorporates a semantic-phonologic approach, but also lexical learning strategies, such as asking for the meaning of a word or the name of an unfamiliar object. It has had a positive impact on receptive vocabulary in 9-year-old children (Motsch & Marks, 2015) indicating that, in addition to using a semantic-phonologic approach, also teaching lexical learning strategies seems to be a promising way to support receptive vocabulary.

The interventions focusing on the comprehension of grammar had mainly positive results on the language skills of school-aged children and adolescents. This aligns with previous research: there are interventions indicating efficacy to improve grammar of school-age children (Ebbels, 2014). One of the most researched techniques, The SHAPE CODING system, indicated promising results on the comprehension of grammar, even with very large effect sizes. However, for some structures (e.g. datives), children with poor auditory memory may show limited progress (Ebbels, 2007). A study on another method called ‘MetaTaal’, using explicit teaching of grammar with visual support, found no effects on oral language comprehension (Zwitserlood et al., 2015). The basic idea of the intervention has similarities with The SHAPE CODING system. It remains unclear why MetaTaal showed no positive results on oral language comprehension, but The SHAPE CODING system did. One possible explanation may be the assessment method used. In the study of Zwitserlood et al. (2015), children had to choose the correct picture from a set of four. A multiple-choice picture-matching task seems to be problematic for assessing comprehension in that it tests skills beyond those of linguistic competence (Frizelle et al., 2019). It is therefore possible that, with other means of assessment, improvements in oral language comprehension following intervention with MetaTaal could have been found.

Teaching of story grammar and using narratives had positive effects on narrative comprehension skills in one study (Petersen et al., 2008). In another study targeting narrative comprehension (Joffe et al., 2019), the narrative comprehension was not measured independently, but a narrative checklist was used, which requires both comprehension of narrative and expressive language skills. The participants, especially in the narrative group, improved in their skills measured with the checklist. This indicates that practising understanding and telling of stories may improve narrative comprehension. A meta-analysis examining instruction designed to foster young children’s narrative skills detected a medium effect size on narrative comprehension (Pesco & Gagné, 2015). More research on the efficacy of scaffolding narratives in school-age children and adolescents is needed, but it seems that scaffolding narratives, for example by teaching story grammar, may be a feasible way of supporting narrative comprehension in this age group.

In the only study *modifying the communicative environment,* an SLT and teachers worked together to modify the language used in the classroom (Starling et al., 2012). A medium effect size on the pupils’ oral language comprehension skills was detected. This aligns with what is known about effective evidence-based professional development of teachers. Sustained and site-based professional development interventions that were conducted by experts resulted in the most positive effects on student outcomes (Guskey & Yoon, 2009). Nonetheless, further research on the efficacy of modification of communicative environment on oral language comprehension of school-age children and adolescents is still needed.

The studies with the least evidence of efficacy aimed at improving *language processing* either by improving auditory temporal processing or aiming at automatisation of specific skills. This aligns with previous research on interventions aiming to improve language processing in some way – so far they have had no positive and lasting effect on oral language skills, including comprehension (Melby-Lervåg & Hulme, 2013; Strong et al., 2011). While direct enhancement of language processing skills does not seem to improve oral language comprehension, using compensatory strategies, such as visualisation, to function with current language processing skills seems to indicate efficacy in improving oral language comprehension. In the present review, two studies indicated that creating mental images may have a positive effect on oral language comprehension (Dixon et al., 2001; Joffe et al., 2007). This aligns with other studies reporting that the use of visualisation might indeed aid oral language comprehension (Center et al., 1999; Oakhill & Patel, 1991). More research on the matter is still needed.

Intensity, frequency, and duration of the interventions varied greatly between the studies included in the present review resulting in a variety of total intervention hours. The literature on dosage is ambiguous in what are optimal intensity, frequency, and duration of an intervention to maximise efficacy. It seems that interventions carried out for 8 weeks or longer are more effective than those carried out for less than 8 weeks (Law et al., 2004). A more intensive or greater amount of treatment has also been linked to clinically significant effect sizes (Schooling et al., 2010). On the other hand, it has been suggested that high frequency and high dose do not always lead to better results: high frequency and low dose or low frequency and high dose in treatment provide better outcomes than interventions where children received high-frequency–high-dose or low-frequency–low-dose treatment (Schmitt, Justice, et al., 2017). The most recent systematic review on dosage suggests that there is a point after which there are diminishing returns from additional dosage (Frizelle et al., 2021). Also, if dose is high session frequency can be reduced. However, further research is required before integrating these findings into clinical practice. In the present review, the dosage of the intervention was not a primary interest. However, it seemed that the dosage was generally not the primary explaining factor of efficacy, as a high number of total intervention hours did not always predict efficacy. In intervention studies using Fast ForWord Language, total intervention hours were around 50, which are the highest number of hours of therapy in the included studies in the present review. Still, Fast ForWord indicated little efficacy on language skills of the participants (Cohen et al., 2005; Fey et al., 2011; Friel-Patti et al., 2001; Gillam et al., 2008). This aligns with a systematic meta-analytic review (Strong et al., 2011) which indicates that Fast ForWord has no effect on children’s oral language. Thus, the efficacy of an intervention seems to be primarily a question of an appropriate choice of therapy technique with a solid theoretical base, and secondarily about the implementation, such as intervention dosage.

When interpreting the results it should be noted that the outcome measure used has an effect on the efficacy. In the present review researcher-created outcome measures examining the targeted skills were more likely to detect a change than standardised tests in the included studies. The clinical tests may show an effect only after a more substantial learning as it often requires generalisation of skills. Effect sizes are also likely to be larger in researcher-created tasks than in clinical tests. In light of this, the following interventions included in the present review seem to indicate efficacy most confidently as the effects are detected with clinical tests: exposure and repetition, identification, and scaffolded manipulation activities (Balthazar & Scott, 2018), narrative-based language intervention (Petersen et al., 2008), and modification of teacher’s language (Starling et al., 2012).

Maintenance of the results in the included studies was high. Maintenance was not always reported, but when it was, the progress was maintained well (see Tables 3 [Table table4-23969415211010423]to 5). The positive improvements in the individual’s skills that originated from the intervention were still evident after weeks or months. Generalisation was also reported in some studies (see Tables 3 [Table table4-23969415211010423]to 5). The results on generalisation indicated that skills learned during the interventions generalised to untrained conditions. It seems that in interventions indicating efficacy, the results are long lasting and generalisation also occurs. However, the number of studies reporting maintenance (38%) and generalisation (33%) were relatively low and this should be considered when interpreting the results.

Of the included studies, only 33% reported effect sizes. They varied from small to very large. A further 24% of the studies reported statistically significant benefits but stated no effect size. Because of the small number and large variation of reported effect sizes, the results can hardly be compared to a suggested benchmark of intervention effects. This suggested benchmark indicates that in children with language disorders, during one academic year while receiving language therapy in public schools, the expected effect size is g=0.51–0.70, that is, a medium effect size (Schmitt, Logan, et al., 2017). The age group in the suggested benchmark are children 3–9 years-of-age. We are not aware of a suggested benchmark for school-age children and adolescents although one is warranted.

To conclude, there are oral language comprehension interventions indicating efficacy in school-age children and adolescents. This aligns with the findings on oral language comprehension interventions for children aged 8 years and younger (Tarvainen et al., 2020). However, it seems that interventions used for improvement of oral language comprehension in children aged 8 years and younger indicate efficacy more often than interventions for school-age children and adolescents: efficacy was indicated in 80% of interventions for children 8 years and younger (Tarvainen et al., 2020) and in 57% for school-age children and adolescents. This difference in the efficacy is likely due to the fact that language processing was attempted to be improved more often in older children than in young children, with no results. When the interventions targeting improvement in language processing (present review n=6; Tarvainen et al., 2020 n=1) are excluded, the respective percentages of interventions indicating efficacy are 83% and 80%. Thus, though learning and therapy techniques used differ between young children and school-age children and adolescents, the results of the present review and those of the previous one (Tarvainen et al., 2020) indicate that both age groups are able to benefit from oral language comprehension interventions.

### Level of evidence

The level of evidence, and thus the confidence one can have in the results of the included studies, varied from high to moderate and to indicative. At present, there are only a few interventions providing high confidence on the efficacy of the intervention methods in question. The most interventions indicating efficacy provided moderate to indicative evidence concerning the results. The level of evidence in relation to treatment efficacy is modest and more research is urgently needed to reach a higher level of evidence for different therapy techniques on oral language comprehension interventions in school-age children and adolescents, and to gain more confidence in the results. One must therefore be cautious when interpreting the results of the present review. While the level of evidence is still partly indicative, other means are also needed to help in choosing appropriate therapy techniques, which is especially true regarding those with the weakest level of evidence. One of the ways to help in decision making in a clinical context is understanding the mechanisms of intervention and being able to specify why and how a therapy works or not (Saldaña & Murphy, 2019), that is, understanding the theoretical underpinnings of the intervention. The different theoretical frameworks of language learning affect the orientation in intervention. For example, targeting automatisation (Hsu & Bishop, 2014) can be seen to represent a behaviourist interpretation of language development as the intervention consisted of drilling of concepts with an extrinsic reward system i.e. errorless learning with a visible reward to correct answers. To give another example, teaching the rules of grammar can be seen to present nativist linguistic theory and Chomskyian grammar (Ebbels, 2007; Ebbels et al., 2014; Ebbels & Lely van der, 2001; Levy & Friedmann, 2009). Teaching the rules of grammar also corresponds well with the Procedural Deficit Hypothesis (Ullman & Pierpont, 2005). The Procedural Deficit Hypothesis states that a possible explanation to language impairment are the deficits in procedural memory. This indicates that the use of declarative memory is required for learning and the children with language impairment should therefore be supported by explicit teaching. Although the theories of language acquisition have a long history, developing the theories of treatment in DLD is still in its infancy (Saldaña & Murphy, 2019) and there is a need to increase our knowledge on the topic. The final decision on therapy techniques should be conducted by combining the knowledge on level of evidence and the theory of intervention to clinical expertise and client values.

### Limitations

The following limitations should be acknowledged when interpreting the results of the present study. In general, it should be noted that the number of studies is small considering the relatively large age and intervention scope. First, factors related to search parameters and inclusion criteria may have affected the results. It is possible that some studies were excluded because the search parameters were not mentioned in the title or in the abstract. Further, studies with various research designs were included in the present review. The inclusion of studies with different research designs increases the number of included studies and thus broadens the view on the matter. At the same time, however, the risk of bias increases. The confidence one can have in the results was examined by evaluating the level of evidence in the present review. This informs the reader also of the possibility of bias in the included studies. However, we acknowledge that the risk of bias in individual studies was not thoroughly examined with the level of evidence categorisation. Further, the time limitation to include only studies from 1996 or later may have excluded some that may have been relevant but published earlier. It was decided, however, that focusing on the studies published during the last 25 years would provide a relevant overall picture on the matter.

Second, factors related to the included articles should be considered when interpreting the results. Only 38% (8/21) of the studies reported maintenance and another 33% (7/21) generalisation. The risk of bias and how the researchers tried to minimise it was reported in 19% (4/21) of the studies and only 10% (2/21) reported the participants’ experiences related to their own skills after the intervention was collected. Further, the effect of the intervention on participation or in everyday life was not evaluated in any of the studies. This indicates that the long-term results of the interventions, generalisation of skills, and impact on the individual’s life remain obscure. In future research, the maintenance and generalisation of skills acquired during the intervention will hopefully receive more emphasis. Examining the experiences of the participants in how they see their improvement and participation is also warranted. A more thorough investigation of the risk of bias would also be reasonable.

### Implications

The results of this systematic scoping review suggest that there are therapy techniques with which oral language comprehension difficulties of 5–16-year-old children with DLD can be ameliorated. However, not all interventions indicate efficacy and the level of evidence is still largely indicative. Though not all interventions aimed at improving oral language comprehension indicate efficacy, there are therapy techniques which focus on different aspects of language and indicate efficacy. A careful choice of therapy techniques is required to support oral language comprehension of school-age children and adolescents with difficulties in oral language comprehension and to minimise risks associated with persistent linguistic difficulties. Interventions indicating efficacy targeted aspects of language or modified the communicative environment. The present findings do not support the use of therapy techniques aiming to improve oral language comprehension by targeting language processing skills. Strategies and compensatory means indicate efficacy in functioning better with current language processing skills, however.

### Further research

There is an evident need for oral language comprehension intervention research in school-age children and adolescents with DLD. Large randomised controlled trials are needed to verify the efficacy of different therapy techniques. When enough studies of different therapy techniques have been conducted, systematic reviews and meta-analyses of the interventions should be executed. Studies using time-series design are also needed to examine the individual patterns of benefits gained from the intervention in relation to different therapy techniques.

Individual topics in need of further research include the relation between dosage and efficacy on oral language comprehension interventions, the efficacy of explicit and implicit therapy techniques in relation to the age of the child, as well as compensatory means and strategies to help individuals function with their persistent language difficulties. In addition, further research is needed on targeting pragmatics to improve oral language comprehension in different contexts. Research on intervention characteristics related to the efficacy of oral language comprehension interventions is also needed. It is important to know what explains the large variability in the efficacy of oral language comprehension interventions. The characteristics related to interventions indicating efficacy should therefore be identified. Better understanding of the mechanisms of efficacy in oral language comprehension is needed to maximise outcomes for individuals with DLD.

The assessment methods used included mostly clinical tests and researcher-created tasks, that is, ways that the clinician assessed the impact of an intervention. Only in 2 of the 21 studies, were the children or adolescents themselves asked whether their skills had improved during the intervention. None of the interventions involved asking about the children’s or adolescents’ experiences regarding how the intervention had impacted their abilities to function in their lives or to participate in everyday situations. The experiences of the children and adolescents are, however, crucial if the goal is to determine the actual impact of the intervention on the individual’s life, i.e. what the clinical significance of the intervention is. There is a need to develop assessment methods to be used by children and adolescents, with possible assistance by adults, in oral language comprehension intervention studies and to examine the characteristics of these assessments.

## Conclusions

The results of the present review indicate high confidence in improving oral language comprehension skills of school-age children and adolescents with DLD with a few carefully chosen therapy techniques. Moderate and indicative level of evidence exists on the efficacy of several other therapy techniques. Interventions targeting aspects of language, compensating current language processing skills, and modifying the communicative environment indicate efficacy, though more research with higher level of evidence is urgently needed. The present results expand the positive findings on oral language comprehension interventions in children aged 1–8 years with language disorders or difficulties (Tarvainen et al., 2020). Further research is obligated on the promising interventions to improve the future prospects of school-age children and adolescents with DLD manifesting in oral language comprehension difficulties. The present findings direct future research and provide information to clinical practice in speech and language therapy.
